# Identification of compound heterozygous *KCNJ1* mutations (encoding ROMK) in a kindred with Bartter's syndrome and a functional analysis of their pathogenicity

**DOI:** 10.1002/phy2.160

**Published:** 2013-11-19

**Authors:** Shalabh Srivastava, Dimin Li, Noel Edwards, Ann-M Hynes, Katrina Wood, Mohamed Al-Hamed, Anna C Wroe, David Reaich, Shabbir H Moochhala, Paul A Welling, John A Sayer

**Affiliations:** 1 Institute of Genetic Medicine, Newcastle UniversityCentral Parkway, Newcastle upon Tyne, NE1 3BZ, U.K.; 2 Newcastle Hospitals NHS Foundation TrustNewcastle upon Tyne, NE7 7DN, U.K.; 3 Department of Physiology, University of Maryland Medical School655 W Baltimore Street, Baltimore, 21201, Maryland; 4 Department of Genetics, King Faisal Specialist Hospital and Research CentreRiyadh, Saudi Arabia; 5 South Tees NHS Foundation TrustMiddlesbrough, TS4 3BW, U.K.; 6 UCL Centre for Nephrology, Royal Free HospitalPond Street, London, NW3 2QG, U.K.

**Keywords:** Hypercalciuria, hypokalemia, KCNJ1, Kir1.1, missense mutation, potassium, ROMK, salt wasting

## Abstract

A multiplex family was identified with biochemical and clinical features suggestive of Bartter's syndrome (BS). The eldest sibling presented with developmental delay and rickets at 4 years of age with evidence of hypercalciuria and hypokalemia. The second sibling presented at 1 year of age with urinary tract infections, polyuria, and polydipsia. The third child was born after a premature delivery with a history of polyhydramnios and neonatal hypocalcemia. Following corrective treatment she also developed hypercalciuria and a hypokalemic metabolic alkalosis. There was evidence of secondary hyperreninemia and hyperaldosteronism in all three siblings consistent with BS. Known BS genes were screened and functional assays of ROMK (alias KCNJ1, Kir1.1) were carried out in *Xenopus* oocytes. We detected compound heterozygous missense changes in *KCNJ1*, encoding the potassium channel ROMK. The S219R/L220F mutation was segregated from father and mother, respectively. *In silico* modeling of the missense mutations suggested deleterious changes. Studies in *Xenopus* oocytes revealed that both S219R and L220F had a deleterious effect on ROMK-mediated potassium currents. Coinjection to mimic the compound heterozygosity produced a synergistic decrease in channel function and revealed a loss of PKA-dependent stabilization of PIP_2_ binding**.** In conclusion, in a multiplex family with BS, we identified compound heterozygous mutations in *KCNJ1*. Functional studies of ROMK confirmed the pathogenicity of these mutations and defined the mechanism of channel dysfunction.

## Introduction

Bartter's syndrome (BS) is a salt-losing tubulopathy which is characterized by hypokalemia, metabolic alkalosis, hyperreninemia, and normotension/low blood pressure (Sayer and Pearce 2001). Additional features may include growth retardation, learning difficulties, hypercalciuria (leading to nephrocalcinosis), and hypomagnesemia. Historically BS has been divided clinically into subtypes including classic BS and antenatal BS. Molecular genetics now allows subtypes of BS to be distinguished (Sayer and Pearce 2001). Mutations in *SLC12A1* (encoding NKCC2) (Simon et al. 1996a,b), *KCNJ1* (encoding ROMK) (Simon et al. 1996a,b), *CLCNKB* (encoding CLC-Kb) (Simon et al. 1997), *CLCNKA* (encoding CLC-Ka) (Schlingmann et al. 2004), *BSND* (encoding Barttin) (Birkenhager et al. 2001), and *CASR* (encoding CaSR) (Watanabe et al. 2002) may all cause a BS phenotype. BS remains rare with an estimated prevalence of 1 in 1,000,000 (Ji et al. 2008). The carrier rate for heterozygous changes in genes associated with salt wasting (Bartter's and Gitelman's syndrome) is estimated to be ∼1%, conferring protection against hypertension in these individuals (Ji et al. 2008). We report clinical, genetic, and functional data from a Pakistani kindred with three affected female members with nephrocalcinosis and biochemical features typical of BS. Using *in silico* and in vitro studies, we identified the molecular and physiological defects underlying this disease phenotype, thereby providing new insights into BS.

## Materials and Methods

Following informed consent, DNA was extracted from peripheral blood cells using standard techniques. Clinical phenotypes including biochemical, histological, and radiological data were reviewed.

To identify chromosome aberrations and regions of homozygosity within this multiplex family, we carried out a genome-wide scan using Affymetrix Cytogenetics Whole-Genome 2.7M Arrays (http://www.affymetrix.com). The data were analyzed using the Chromosome Analysis Suite (ChAS) software (Affymetrix UK Ltd., High Wycombe, U.K.). Mutational screening was undertaken of known genes implicated in BS, including *KCNJ1*, *CLCKNB*, *SLC12A1*, *BSND*, and *CASR*. Direct sequencing of all coding exons and exon–intron boundaries was performed. Polymerase chain reaction (PCR) products were sequenced using BigDye Terminator Cycle Sequencing kit (PE Applied Biosystems, Beverly, MA). Sequences were analyzed using Mutation Surveyor software Version 3.24 (SoftGenetics LLC, State College, PA). Computational analyses of novel missense mutations were performed with PolyPhen-2 (http://genetics.bwh.harvard.edu/pph2/). PolyPhen-2 uses eight sequence-based and three structure-based predictive features and scores range from 0 to 1; the higher the score the more damaging the amino acid substitution (Adzhubei et al. 2010). A control DNA panel from 96 healthy individuals was used to screen for sequence variants.

### Homology modeling human ROMK

Human ROMK (NP_000211) was modeled using the crystal structure of chicken Kir2.2/Kcnj12 (Protein Data Bank code, 3SPC) (Hansen et al. 2011). ROMK and Kcnj12 amino acid sequences were aligned using HHPred (Soding 2005) and a three-dimensional model generated using Modeller (Sali and Blundell 1993). Figures were prepared using PyMOL (http://www.pymol.org/).

### Subcloning, mutagenesis, and *Xenopus* oocyte injection

Full-length human ROMK transcript variant 1 (NM_000220) was subcloned into vector pSD64TR. Site-directed mutagenesis was performed to introduce mutations S219R and L220F. Mutated KCNJ1 constructs were Sanger sequenced in their entirety to confirm mutation, orientation, and fidelity (data not shown).

### cRNA synthesis

Complementary RNA was transcribed in vitro in the presence of capping analog from linearized plasmids using SP6 RNA polymerase (mMessage Machine; Ambion Inc., Life Technologies, Carlsbad, CA). cRNA was purified by spin column chromatography (MEGAclear; Ambion Inc.). Yield was quantified spectrophotometrically and confirmed by agarose gel electrophoresis.

### Functional characterization of ROMK in *Xenopus* oocytes

*Xenopus laevis* (*Xenopus* Express, Homosassa, FL) oocytes were isolated using a protocol approved by the Institutional Animal Care and Use Committee at the University of Maryland Medical School. Oocyte aggregates were dissected from the ovarian lobes, and then incubated in OR-2 medium (82.5 mmol/L NaCl, 2 mmol/L KCl, 1 mmol/L MgCl_2_, and 5 mmol/L hydroxyethyl piperazineethanesulfonic acid (HEPES), pH 7.5)-containing collagenase (type 3, Worthington) for 2 h at room temperature. Oocytes were stored at 19°C in OR-3 medium (50% Leibovitz's medium and 10 mmol/L HEPES, pH 7.4). Healthy-looking Dumont stage V–VI oocytes were injected with 50 nL of diethylpyrocarbonate-treated water containing 250 pg of ROMK cRNA 12–24 h later, and were then incubated in OR-3 medium at 19°C. Experiments were performed 3 days after injection.

### Electrophysiology

Whole-cell currents in *Xenopus* oocytes were monitored using a two-microelectrode voltage clamp (OC-725; Warner Instruments, LLC, Hamden, CT). Voltage sensing and current injecting microelectrodes had resistances of 0.5–1.5 MΩ when backfilled with 3mol/L KCl. Data were collected using an ITC16 analog-to-digital, digital-to-analog converter (Instrutech Corp., Port Washington, NY), filtered at 1 kHz, and digitized on line at 2 kHz using Pulse software (HEKA Electronik, Pfalz, Germany) for later analysis. Once a stable membrane potential was attained, oocytes were clamped to a holding potential at the predicted potassium equilibrium potential (i.e., near-zero current value) and currents were recorded during 500 msec voltage steps, ranging from −120 mV to +60 mV in 20 mV increments. ROMK potassium currents are taken as the barium-sensitive current (1 mmol/L Ba acetate). Oocytes were bathed in a 90 mmol/L KCl solution (5 mmol/L KCl, 85 mmol/L *N*-methyl-d-glucamine-gluconate, 1 mmol/L MgCl_2_, 1 mmol/L CaCl_2_, and 5 mmol/L HEPES, pH 7.4) and outward currents at 0 mV are reported. To study M1 receptor-dependent regulation of ROMK, outward potassium currents (at 0 mV) were measured before and after the addition of carbachol (25 *μ*mol/L, 10 min). Data were analyzed by one-way analysis of variance (ANOVA) with Tukey–Kramer posttesting using GraphPad Prism v.4 (GraphPad Software, San Diego).

## Results

**Figure 1:**
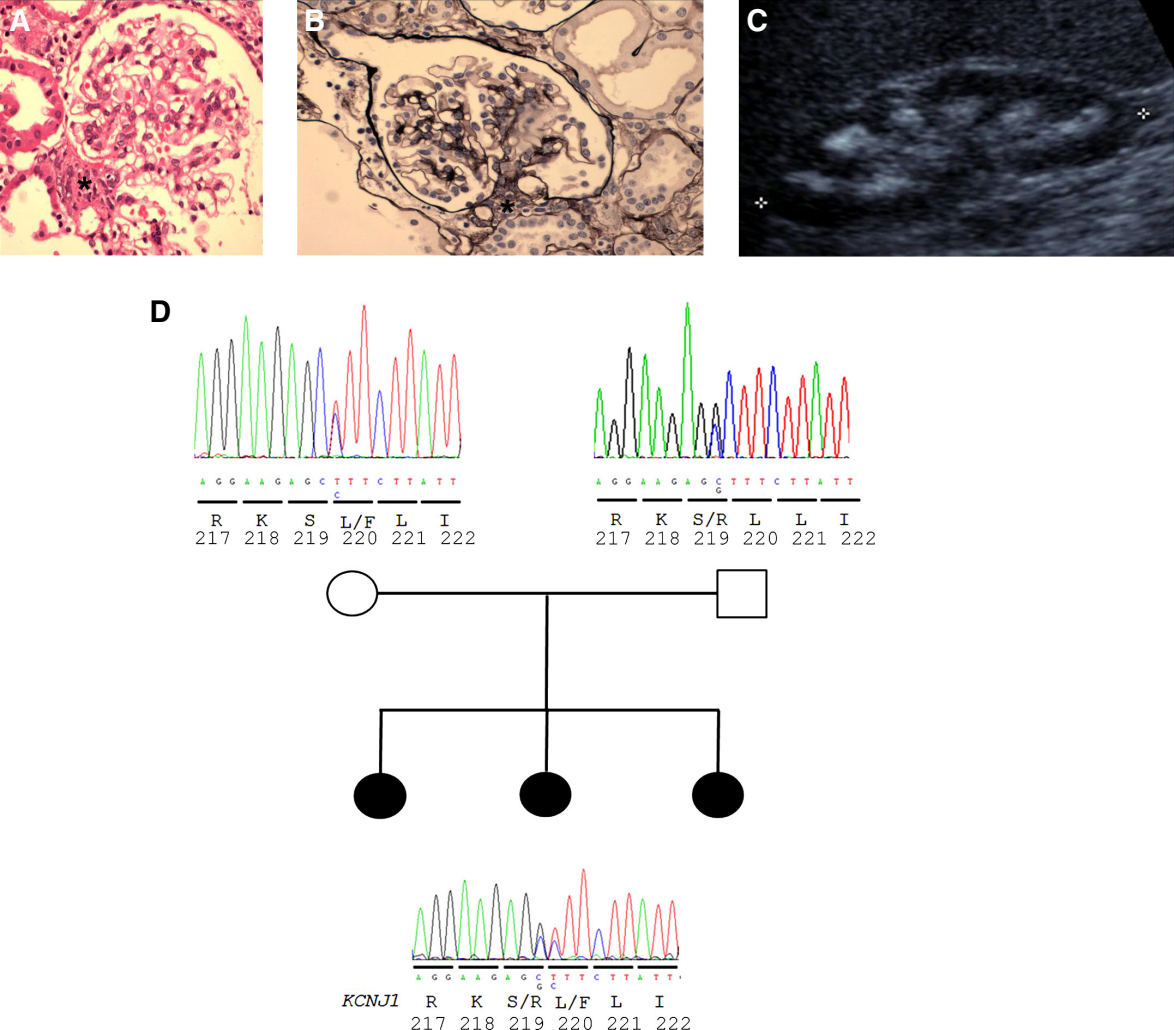
Clinical and molecular genetic analysis of Bartter's syndrome patients. Hyperplasia of juxtaglomerular apparatus is demonstrated on renal histology in (A) sibling 1 and (B) sibling 2. (C) Renal USS demonstrates nephrocalcinosis in sibling 3. (D) *KNCJ1* sequencing identifies compound heterozygous mutations in all three affected p.S219R/p.L220F segregating from each parent.

We identified a multiplex Pakistani family with three affected individuals with a salt-wasting tubulopathy. A summary of the clinical and biochemical findings is given in Table 1. The eldest sibling presented with developmental delay following a routine check and an abnormal gait at 4 years of age. She was below the 3rd centile for both weight and height. Her father reported polydipsia and radiography examination confirmed rickets. She was slightly hypocalcemic (2.05 *μ*mol/L [reference range 2.1–2.6]) and had a mild hypokalemic metabolic alkalosis. Following treatment with vitamin D supplements, she developed hypercalciuria and had evidence of nephrocalcinosis on renal ultrasound scanning. This was treated with a combination of hydrochlorothiazide and potassium chloride supplementation. At 9 years of age, declining renal function prompted a percutaneous renal biopsy (Fig. 1A).

**Table 1:** Clinical features of affected individuals and their parents

	Sibling 1	Sibling 2	Sibling 3	Father	Mother
Age at presentation	4 years	1 year	1 year	N/A	N/A
Polyhydramnios	Unknown	Y	Y	N	N
Polydipsia	Y	Y	Y	N	N
Sensorineural deafness	N	N	N	N	N
Learning difficulties	Y	Y	Y	N	N
Serum potassium (μmol/L) (NR 3.5–5.3)	2.5–3	3.9	2.5–3	4.4	3.8
Serum bicarbonate (μmol/L) (NR 22–29)	30	25	23	22	23
Serum calcium (μmol/L) (NR 2.12–2.6)	2.05	1.89	1.85	2.4	2.35
Serum magnesium (mmol/L) (NR 0.7–1.0)	0.68	0.54	0.64	N/A	N/A
Serum creatinine (μmol/L) (NR 55–95)	65	117	54	42	78
Renin (nmol/L h^−1^) (NR 2.8–4.5)	>50	3.6	N/A	N/A	N/A
Aldosterone (pmol/L) (NR 100–450)	345	5665	N/A	N/A	N/A
Urine calcium:creatinine ratio (mmol/mmol creatinine) (NR 0–0.7)	1.25	1.41	3.2	Not done	Not done
Renal biopsy	Occasional foci of calcification around tubules JGA hyperplasia	Occasional foci of calcification around tubules JGA hyperplasia	Not biopsied	Not biopsied	Not biopsied
Renal ultrasound scan	Nephrocalcinosis	Nephrocalcinosis	Nephrocalcinosis	Not performed	No nephrocalcinosis
Systolic blood pressure (mmHg)	Normotensive 80–100	Normotensive 70–80	Normotensive 70–80	Normotensive 130	Normotensive 120

NR, normal range; N/A, not available.

The second sibling had documented polyhydramnios and low birth weight (2.6 kg). Childhood urinary tract infections at 1 year of age prompted further investigations revealing polyuria, polydipsia, and hypercalciuria. By 4 years of age there was radiological evidence of rickets and nephrocalcinosis, and documented learning difficulties and hypokalemia. The child was also treated with hydrochlorothiazide and potassium chloride supplementation. This child also underwent renal biopsy in the context of an acute decline in renal function (Fig. 1B).

The third sibling was born prematurely with a history of maternal polyhydramnios. Hypercalciuria was severe, leading to medullary nephrocalcinosis and suggesting an underlying metabolic cause (Fig. 1C). There were no other unaffected siblings and the clinical and biochemical phenotype of the parents was normal (Table 1).

The combined clinical and biochemical picture of the three affected siblings (Table 1 and Fig. 1) suggested a clinical diagnosis of BS. There is considerable genetic heterogeneity within BS and we wondered if the biochemical picture reflected a specific genotype. The hypocalcemia exhibited by two of the siblings together with a BS phenotype suggested that a *CASR* mutation may be the underlying cause (Sayer and Pearce 2003). There was no history of sensorineural deafness, making *BSND* mutations unlikely; however, the antenatal presentation of two of the siblings (with polyhydramnios) suggested a *SLC12A1* or *KCNJ1* mutation.

However, given this uncertainty and the genetic heterogeneity of BS (six genes and many exons) and because of possible parental consanguinity, we initially performed homozygosity mapping of the three affected individuals and their parents. This, however, did not reveal any large regions of homozygosity by descent (data not shown). We therefore proceeded to examine, using exon PCR and Sanger sequencing, known genes for BS. Genes *SLC12A1*, *KCNJ1*, *CLCNKB*, *BSND*, and *CASR* were screened. We detected compound heterozygous missense changes in *KCNJ1* gene, encoding the renal outer medullary potassium channel ROMK. The p.S219R/p.L220F mutation segregated from father and mother, respectively (Fig. 1D).

*In silico* modeling revealed that the missense mutations were both likely to have deleterious effects on channel function. PolyPhen-2 predicts that the S219R change is probably damaging (with a score of 0.999) and that the L220F change is probably damaging (with a score of 1.0). Both scores can be interpreted as a confident prediction of a pathogenic amino acid change.

**Figure 2:**
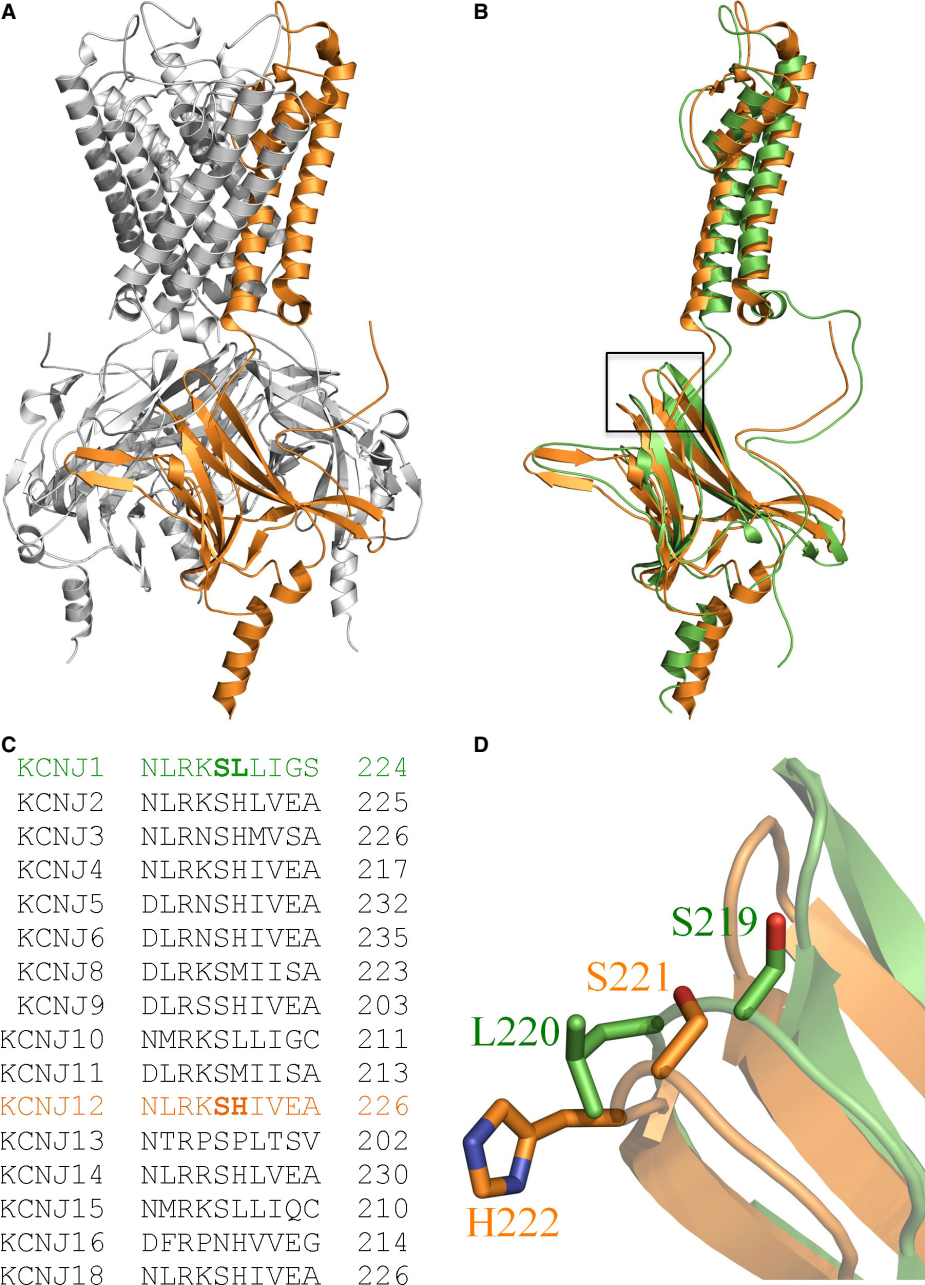
Homology modeling of ROMK. (A) Crystal structure of chicken Kcnj12/Kir2.2 (Hansen et al. 2011). One of the four Kcnj12 monomers forming the potassium channel is highlighted in orange. (B) Putative structure of the ROMK monomer (green) compared with chicken Kcnj12 (orange). The black box denotes the position of S219 and L220 in ROMK. (C) Partial amino acid sequence alignment of the human KCNJ family. (D) Putative position of S219 and L220 in ROMK and the homologous residues in chicken Kcnj12 (S221 and H222, respectively).

Modeling of the ROMK protein (Fig. 2) was undertaken exploiting homology to the crystal structure of a related inwardly rectifying potassium channel, Kcnj12/Kir2.2 (Hansen et al. 2011). This predicted a high degree of structural homology between ROMK and Kcnj12.

**Figure 3:**
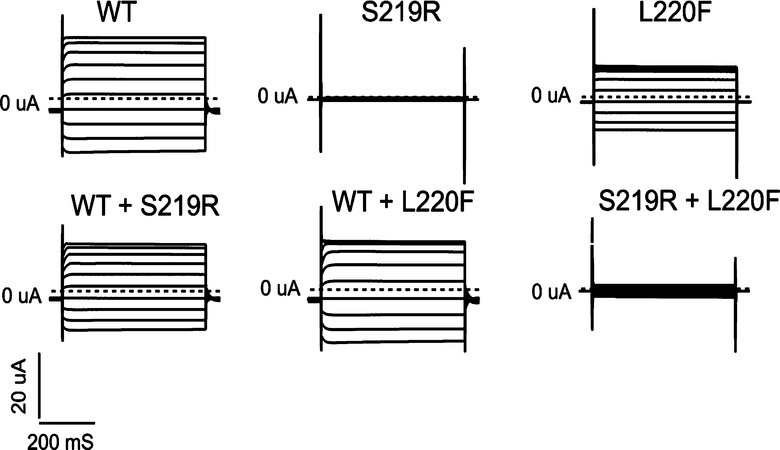
Comparison of the properties of wild-type (WT) and mutant ROMK channels in *Xenopus* oocytes. Shown are families of whole-cell currents in oocytes injected with ROMK cRNA-encoding WT, mutant cRNA (S219R and L220F), or combinations of both to mimic heterozygosity and compound heterozygosity. Oocytes were held at −60 mV and clamped in 20 mV increments from −120 mV to +60 mV in 20 mV steps (5 mmol/L potassium).

**Figure 4:**
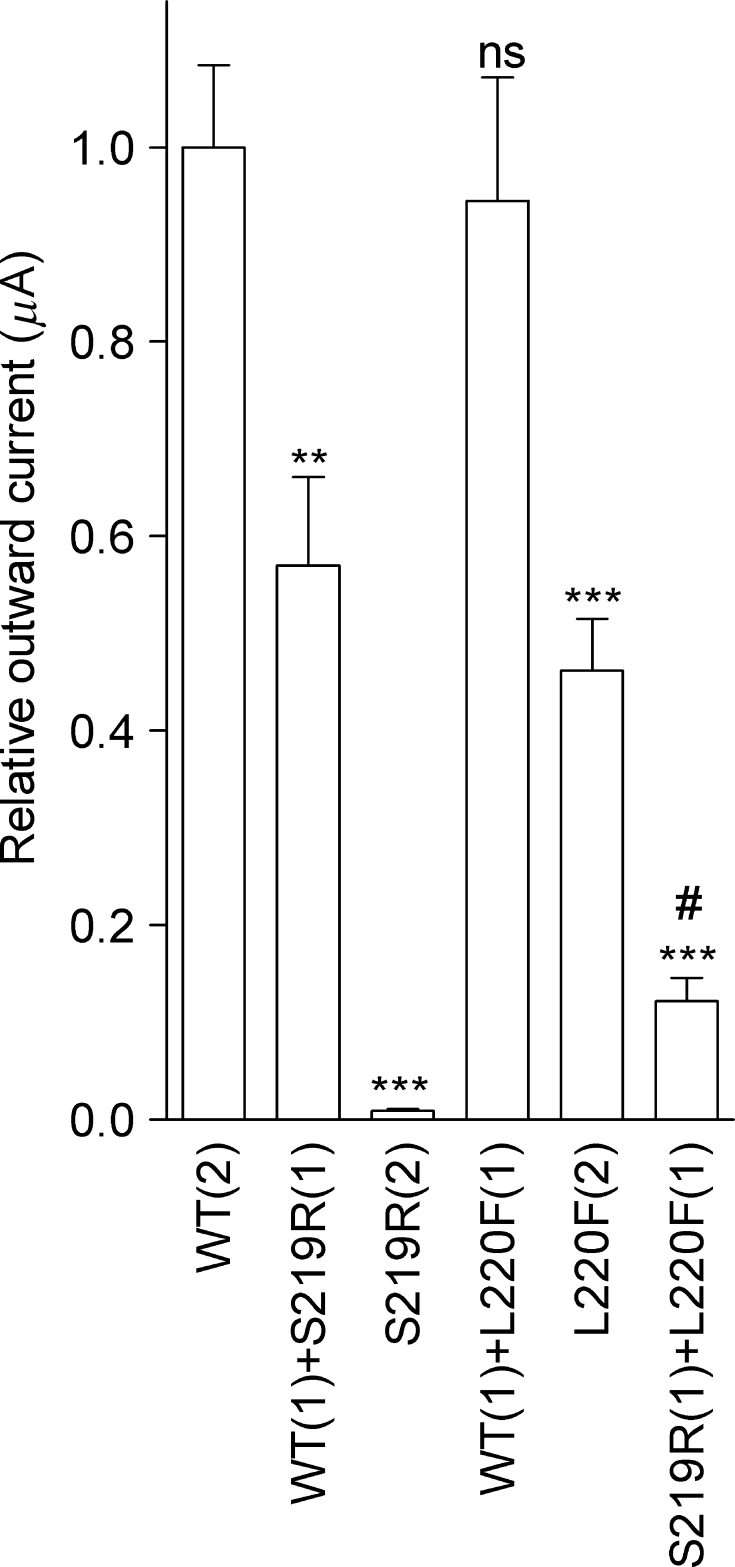
Effect of S219R and L220F mutations on ROMK channel activity. Relative outward potassium currents recorded by two-electrode voltage -clamp in *Xenopus* oocytes expressing ROMK. Oocytes were injected with 250 pg cRNA-encoding wild type (WT), S219R, or L220F mutant channel subunits. To mimic the compound heterozygous state of the patients, oocytes were coinjected with mixtures (given in parentheses) of the different cRNAs (250 pg in total). Data are mean ± SEM (*n* = 18 oocytes/group, from three frogs). ***P < 0.001; **P < 0.01; ns, *P* > 0.05 versus WT potassium current. ^#^*P* < 0.001 versus WT+S219R current.

To test the consequence of the mutations on channel function, biophysical properties of the channels were assessed by two-electrode voltage clamp in *Xenopus* oocytes, expressing or coexpressing wild-type (WT) and mutant ROMK (Figs. 3 and 4). We found that channels homozygous for either mutation (S219R and L220F) exhibited significantly (*P* < 0.001) reduced potassium currents compared to the WT channel (Figs. 3 and 4). The homozygous S219R mutant completely abolished the potassium current, whereas channels homozygous for the L220F mutation carried ∼50% the potassium current as the WT channel. As expected from the recessive nature of the disease, studies in oocytes coinjected with equivalent doses of WT and mutant ROMK (mimicking the heterozygous state of the mother and father) revealed that neither mutation exhibited dominant inhibitory behavior. In fact, the activity of ROMK channels comprising WT and L220F mutant subunits (WT+L220F) was not significantly (*P* > 0.05) different from the WT channel, whereas the potassium current carried by channels comprising WT and S219R subunits (WT+S219R) was reduced by ∼50%. By contrast, when both S219R and L220F mutants were coexpressed together (S129R+L220F) to mimic the compound heterozygous state of the patients, channel activity was almost completely abolished (Figs. 3 and 4). Such synergistic negative effects provide a molecular explanation of the disease.

**Figure 5:**
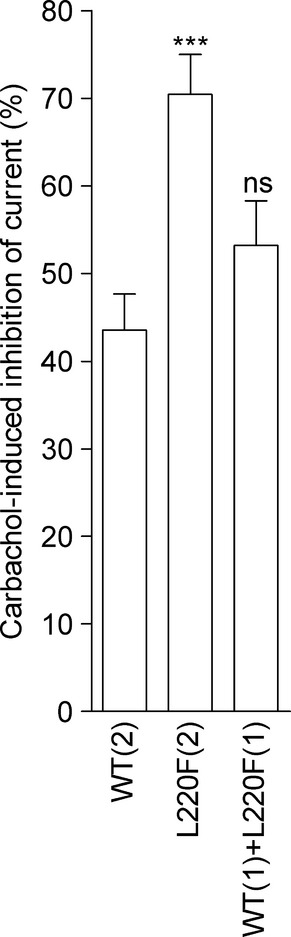
L220F affects phosphatidylinositol 4,5-bisphosphate (PIP_2_)-dependent gating of ROMK. Whole-cell potassium currents recorded in *Xenopus* oocytes coexpressing the M1 receptor with ROMK channels comprising wild type (WT) and/or L220F mutant subunits. Relative doses are given in parentheses. Data are expressed as the percent inhibition of the potassium current induced by exposure (10 min) to carbachol (25 *μ*mol/L) and are mean ± SEM (*n* = 12–15 oocytes/group, from three frogs). ***P < 0.001; ns, *P* > 0.05 versus inhibition of WT potassium current.

ROMK requires protein kinase A (PKA)-dependent phosphorylation (Xu et al. 1996) and binding of phosphatidylinositol 4, 5 biphosphate (PIP_2_) (Huang et al. 1998) to stay open. The S219R mutation is especially damaging because S219 is a key PKA phosphorylation site (Xu et al. 1996). The neighboring L220F mutation may also corrupt the PKA phosphorylation site, albeit to a lesser degree, as a smaller hydrophobic residue is preferred at this position for efficient phosphorylation. As phosphorylation is coupled to PIP_2_-dependent gating (Huang et al. 1998), we explored whether the mutations affected this process. Normally, ROMK gating is relatively resistant to physiological changes in PIP_2_ levels because of the high affinity of PIP_2_ binding (Fang et al. 2010). Because L220F is partially active, we focused on this mutant to explore whether the mutation is sufficient to decrease PIP_2_ affinity. For these studies, we tested whether channels acquire sensitivity to G protein-coupled receptor (GPCR)-mediated changes in PIP_2_ levels by coexpressing ROMK with the M1 muscarinic receptor and measuring outward potassium currents before and after addition of ligand (carbachol, 25 *μ*mol/L). This GPCR activates phospholipase C, which induces PIP_2_ hydrolysis (thereby inhibiting channel activity) and has been used widely to study PIP_2_ regulation of Kir channels, including ROMK (Zeng et al. 2003). Compared to the wild-type ROMK, potassium currents carried by ROMK L220F rapidly declined following application of carbachol (Fig. 5). Thus, it seems likely that the diminished function in heteromeric S219R/L220F channels results from loss of PKA-dependent stabilization of PIP_2_ binding. Of note, the inhibitory effect of carbachol was lost in heteromeric channels formed of L220F and the WT subunits (WT+L220F) as might be expected from the absence of disease in the parents.

## Discussion

Bartter's syndrome is an autosomal recessive disorder leading to salt wasting and a hypokalemic metabolic acidosis. Hypercalciuria is also a typical feature, which leads to the complications of nephrocalcinosis and nephrolithiasis (Sayer and Pearce 2001). A typical presentation would include polyuria and polydipsia, as exemplified by the cases we present. Maternal polyhydramnios, as seen with two of the siblings presented here, and premature labor are also typical features (Sieck and Ohlsson 1984). Salt wasting leads to a chronic activation of the renin–angiotensin–aldosterone axis, and renal biopsy may reveal hyperplasia of the juxtaglomerular apparatus, as originally detailed by Bartter (Bartter et al. 1962). Additional clinical features may include short stature (Buyukcelik et al. 2012), failure to thrive, and developmental delay. Mutations in genes-encoding thick ascending limb of Henle (TALH) transporters NKCC2, ROMK, CLC-Ka, CLC-Kb, and the subunit Barttin may all result in a BS phenotype (Proesmans 2006). The CaSR is expressed on the basolateral membrane of the TALH, and gain of function mutations in *CASR* may also lead to a salt-wasting phenotype in addition to hypocalcemia (Sayer and Pearce 2003). In the present cases, we unexpectedly identified compound heterozygous mutations in *KCNJ1* accounting for the BS phenotype. The molecular genetic analysis allowed the identification of a single-gene disorder despite the complex presentation that included hypocalcemia. The hypocalcemia was likely due to maternal and childhood vitamin D deficiency (Kovacs 2013) and responded to vitamin D supplementation.

The S219R mutation (also previously reported in heterozygous state as S200R by Simon et al. [1996a,b]) is predicted to disrupt a PKA phosphorylation site, which has been shown to be important to channel function (Xu et al. 1996). The L220F mutation has previously been identified (in its heterozygous state) in a child with neonatal BS (Vollmer et al. 1998). *In silico* analysis pointed toward a pathogenic effect of both missense variants, and electrophysiological studies established convincingly that both S219R and L220F mutations reduced potassium currents. Interestingly, we were able to mimic the compound heterozygous changes in ROMK. By expressing both S219R and L220F mutants together (S129R+L220F) the channel activity was severely impaired. In order to investigate the L220F mutation further, we explored the effect of PIP_2_ regulation of ROMK channel activity. Compared to the wild-type ROMK, potassium currents carried by ROMK L220F rapidly declined following PIP_2_ hydrolysis. Thus, we provide convincing evidence that reduced function in heteromeric S219R/L220F ROMK channels results from loss of PKA-dependent stabilization of PIP_2_ binding.

## References

[b1] Adzhubei IA, Schmidt S, Peshkin L, Ramensky VE, Gerasimova A, Bork P (2010). A method and server for predicting damaging missense mutations. Nat. Methods.

[b2] Bartter FC, Pronove P, Maccardle JR, Gill RC (1962). Hyperplasia of the juxtaglomerular complex with hyperaldosteronism and hypokalemic alkalosis. A new syndrome. Am. J. Med.

[b3] Birkenhager R, Otto E, Schurmann MJ, Vollmer M, Ruf EM, Maier-Lutz I (2001). Mutation of BSND causes Bartter syndrome with sensorineural deafness and kidney failure. Nat. Genet.

[b4] Buyukcelik M, Keskin M, Kilic BD, Kor Y, Balat A (2012). Bartter syndrome and growth hormone deficiency: three cases. Pediatr. Nephrol.

[b5] Fang L, Li D, Welling PA (2010). Hypertension resistance polymorphisms in ROMK (Kir1.1) alter channel function by different mechanisms. Am. J. Physiol. Renal Physiol.

[b6] Hansen SB, Tao X, MacKinnon R (2011). Structural basis of PIP_2_ activation of the classical inward rectifier K^+^ channel Kir2.2. Nature.

[b7] Huang CL, Feng S, Hilgemann DW (1998). Direct activation of inward rectifier potassium channels by PIP_2_ and its stabilization by Gbetagamma. Nature.

[b8] Ji W, Foo JN, O'Roak BJ, Zhao H, Larson MG, Simon DB (2008). Rare independent mutations in renal salt handling genes contribute to blood pressure variation. Nat. Genet.

[b9] Kovacs CS (2013). Maternal vitamin D deficiency: fetal and neonatal implications. Semin. Fetal Neonatal. Med.

[b10] Proesmans W (2006). Threading through the mizmaze of Bartter syndrome. Pediatr. Nephrol.

[b11] Sali A, Blundell TL (1993). Comparative protein modelling by satisfaction of spatial restraints. J. Mol. Biol.

[b12] Sayer JA, Pearce SH (2001). Diagnosis and clinical biochemistry of inherited tubulopathies. Ann. Clin. Biochem.

[b13] Sayer JA, Pearce SH (2003). Extracellular calcium-sensing receptor dysfunction is associated with two new phenotypes. Clin. Endocrinol. (Oxf.).

[b14] Schlingmann KP, Konrad M, Jeck N, Waldegger P, Reinalter SC, Holder M (2004). Salt wasting and deafness resulting from mutations in two chloride channels. N. Engl. J. Med.

[b15] Sieck UV, Ohlsson A (1984). Fetal polyuria and hydramnios associated with Bartter's syndrome. Obstet. Gynecol.

[b16] Simon DB, Karet FE, Hamdan JM, DiPietro A, Sanjad SA, Lifton RP (1996a). Bartter's syndrome, hypokalaemic alkalosis with hypercalciuria, is caused by mutations in the Na-K-2Cl cotransporter NKCC2. Nat. Genet.

[b17] Simon DB, Karet FE, Rodriguez-Soriano J, Hamdan JH, DiPietro A, Trachtman H (1996b). Genetic heterogeneity of Bartter's syndrome revealed by mutations in the K+ channel, ROMK. Nat. Genet.

[b18] Simon DB, Bindra RS, Mansfield TA, Nelson-Williams C, Mendonca E, Stone R (1997). Mutations in the chloride channel gene, CLCNKB, cause Bartter's syndrome type III. Nat. Genet.

[b19] Soding J (2005). Protein homology detection by HMM-HMM comparison. Bioinformatics.

[b20] Vollmer M, Koehrer M, Topaloglu R, Strahm B, Omran H, Hildebrandt F (1998). Two novel mutations of the gene for Kir 1.1 (ROMK) in neonatal Bartter syndrome. Pediatr. Nephrol.

[b21] Watanabe S, Fukumoto S, Chang H, Takeuchi Y, Hasegawa Y, Okazaki R (2002). Association between activating mutations of calcium-sensing receptor and Bartter's syndrome. Lancet.

[b22] Xu ZC, Yang Y, Hebert SC (1996). Phosphorylation of the ATP-sensitive, inwardly rectifying K^+^ channel, ROMK, by cyclic AMP-dependent protein kinase. J. Biol. Chem.

[b23] Zeng WZ, Li XJ, Hilgemann DW, Huang CL (2003). Protein kinase C inhibits ROMK1 channel activity via a phosphatidylinositol 4,5-bisphosphate-dependent mechanism. J. Biol. Chem.

